# Novel Developmental Analyses Identify Longitudinal Patterns of Early Gut Microbiota that Affect Infant Growth

**DOI:** 10.1371/journal.pcbi.1003042

**Published:** 2013-05-09

**Authors:** Richard A. White, Jørgen V. Bjørnholt, Donna D. Baird, Tore Midtvedt, Jennifer R. Harris, Marcello Pagano, Winston Hide, Knut Rudi, Birgitte Moen, Nina Iszatt, Shyamal D. Peddada, Merete Eggesbø

**Affiliations:** 1 Department of Genes and Environment, Division of Epidemiology, Norwegian Institute of Public Health, Oslo, Norway; 2 Department of Biostatistics, Harvard School of Public Health, Boston, Massachusetts, United States of America; 3 Department of Infectious Disease Epidemiology, Division of Infectious Disease Control, Norwegian Institute of Public Health, Oslo, Norway; 4 Epidemiology Branch, National Institute of Environmental Health Sciences, National Institutes of Health, Department of Health and Human Services, Research Triangle Park, North Carolina, United States of America; 5 Laboratory of Medical Microbial Ecology, Karolinska Institute, Stockholm, Sweden; 6 Norwegian University of Life Sciences, Department of Chemistry, Biotechnology and Food Science, Aas, Norway; 7 The Norwegian Institute of Food Fisheries and Aquaculture, Aas, Norway; 8 Biostatistics Branch, National Institute of Environmental Health Sciences, National Institutes of Health, Department of Health and Human Services, Research Triangle Park, North Carolina, United States of America; University of Chicago, United States of America

## Abstract

It is acknowledged that some obesity trajectories are set early in life, and that rapid weight gain in infancy is a risk factor for later development of obesity. Identifying modifiable factors associated with early rapid weight gain is a prerequisite for curtailing the growing worldwide obesity epidemic. Recently, much attention has been given to findings indicating that gut microbiota may play a role in obesity development. We aim at identifying how the development of early gut microbiota is associated with expected infant growth. We developed a novel procedure that allows for the identification of longitudinal gut microbiota patterns (corresponding to the gut ecosystem developing), which are associated with an outcome of interest, while appropriately controlling for the false discovery rate. Our method identified developmental pathways of *Staphylococcus* species and *Escherichia coli* that were associated with expected growth, and traditional methods indicated that the detection of *Bacteroides* species at day 30 was associated with growth. Our method should have wide future applicability for studying gut microbiota, and is particularly important for translational considerations, as it is critical to understand the timing of microbiome transitions prior to attempting to manipulate gut microbiota in early life.

## Author Summary

Some obesity trajectories are set early in life, with rapid weight gain being a risk factor for later development of obesity. Recently, much attention has been given to findings indicating that gut microbiota may play a role in obesity development. The existence of time-dependent exposure windows, which rely on stimuli from the gut to initiate healthy development, gives the evolution of early life gut microbiota a critical role in human health. We identified children that followed their expected growth trajectories at six months of life, and those that had deviated. We then developed a novel statistical approach that allowed the identification of longitudinal gut microbiota patterns (e.g. a particular species was detected at days 4, 10, and 30 and not detected at day 120) that were associated with expected growth, while appropriately restricting the false discovery rate. We further identified when a deviation from the proposed longitudinal gut microbiota patterns would result in an abnormal growth outcome (either rapid or decreased growth at six months of life). We found developmental pathways of *Staphylococcus* species and *Escherichia coli* that were associated with expected growth, as well as indications that *Bacteroides* species at day 30 was associated with growth.

## Introduction

Gut microbiota has a critical role in human health [1]–[6]; early infancy is of special interest because the early life period is a determinant for the subsequent adult-like microbiota. Once the first microbes arrive in the sterile gut of the newborn, a dynamic process starts, where activation of genes and expression of receptors in the host plays an important role for the building of niches and the further selection of microbes. More importantly, studies on germ free animals have revealed the presence of time-dependent exposure windows that rely on microbial stimuli from the gut [7] (i.e. development of tolerance [8], [9], sensitivity to biogenic amines [10], influences on cecum size [10], and optimal functioning of diverse systems, such as angiogenesis [11] and stress responses [12]).

Obesity has been linked to gut microbiota in humans, by being associated with reduced bacterial diversity and altered representation of bacterial genes and metabolic pathways [4]. Since rapid weight gain in early life is a risk factor for the later development of obesity [13], we aimed to study whether early infant gut microbiota was associated with the World Health Organization's definition of expected growth in the first six months of life. As gut microbiota can be altered, or even transplanted [4], there is large potential for future medical interventions.

We describe a novel method that identifies patterns of gut microbiota exposures associated with potential time-dependent exposure windows in longitudinal data. We implement this method in the Norwegian Microflora Study (NOMIC) to reveal which patterns of gut microbiota (representing the gut ecosystem developing) are associated with expected infant growth, and compare the results to a standard linear regression model.

We aim at identifying how the development of early gut microbiota affects infant growth. Proper knowledge of the time dependencies of gut microbiota as an exposure is a crucial underpinning before experimental attempts to manipulate early gut microbiota can be made. In light of this, our method will have considerable future applications, especially in the translational area of gut microbiota research.

## Materials and Methods

### Ethics statement

The study was approved by the Regional Ethics Committee for Medical Research in Norway (approval ref 2002, S-02216) and the Norwegian Data Inspectorate (ref 2002/1934-2). The approvals, as well as informed consent from the mothers, were obtained prior to collection of data and samples.

### Study population

NOMIC is a birth cohort designed to study the establishment of gut microbiota during infancy and its consequences for child health. Participating mothers were recruited to the NOMIC study by a paediatrician at the maternity ward in a county hospital in South Norway. The recruitment protocol purposefully oversampled preterm children; whenever a preterm-birth mother was enrolled, two mothers of consecutively born term infants were recruited. The recruitment started in November 2002 and was completed in May 2005. Eligibility criteria required that mothers were fluent in Norwegian and a resident in the pertinent geographic area.

After the informed consent forms were signed by the mothers, containers for fecal samples and a questionnaire were provided to the participants at the maternity ward. The mothers were asked to collect and freeze one fecal sample from themselves at postpartum day 4, as well as samples from their infants when they were 4, 10, 30, and 120 days old. Study personnel retrieved the fecal samples and kept them frozen during transport to the Biobank of the Norwegian Institute of Public Health, Oslo, where they were stored at −20 C upon arrival. Further questionnaires were sent to the families when their infants were aged 6, 12, 18, and 24 months.

Six hundred and one mothers agreed to participate in the NOMIC study, however, 86 (14%) of these mothers never returned any fecal samples, which left 524 infants with available fecal samples from one or more occasions. Children that were preterm (152) (defined as gestational age less than 253 days), term children born via caesarean section (90), or term vaginally born children who had been exposed to antibiotics before day 4 of life (36), were then excluded from the current analysis, leaving 246 children.

### Outcome

Mothers extracted information on weight from their “baby health visit” cards and reported this information in questionnaires. Information on gestational age and preterm delivery was obtained from the Medical Birth Registry of Norway.

To be included in the analysis, we required birthweight and another weight measurement within 122 to 244 days of birth (approximately 4 to 8 months). These two measurements are henceforth referred to as measurements at birth and approximately 6 months of life. If multiple measurements were available during the latter period, the closest to 6 months was used. Data from 218 children (110 females and 108 males) met the inclusion criteria.

A child is expected to follow the percentile given by its birth weight, which can be expressed as an age and sex standardised Z-score. Following recommendations from the Norwegian Health Directorate [14], we used the World Health Organisation's weight-for-age growth curves [15] to describe expected growth. These sex-specific growth curves start with birthweight and provide the percentile distribution of infant weights for infants across ages. We generated sex standardised Z-scores for birth (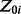) and for six months of age (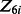), and these Z-scores were compared. The Z-scores were calculated at the time of measurement. We chose approximately 6 months (instead of 12 or 24 months) as the outcome because we had the most complete dataset at this time, and reviews on rapid growth in early childhood failed to differentiate any particular time point from 6 to 24 months as clearly superior in predicting later obesity [13], [16].

We defined the outcome of interest to be the difference in Z-scores: 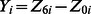. This definition was chosen to be in concordance with the current literature, where the most frequent definition of rapid growth was a Z-score change in weight-for-age [13]. If a child's Z-score deviated between time periods, it was indicative of deviant growth and labelled as either increased growth (reaching higher weights than expected from its birthweight) or decreased growth (undershooting the target weight and reaching lower weights than expected). If a child's growth followed the expected growth trajectory described by the WHO growth curves, they would have an outcome of 0. As is often done in studies focusing on growth of infants, we used the change in Z-score threshold of 0.67 to define expected growth [13]. Thus infants with a Z-score of between 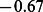 and 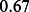 are regarded to be growing as expected.

**Figure 1:**
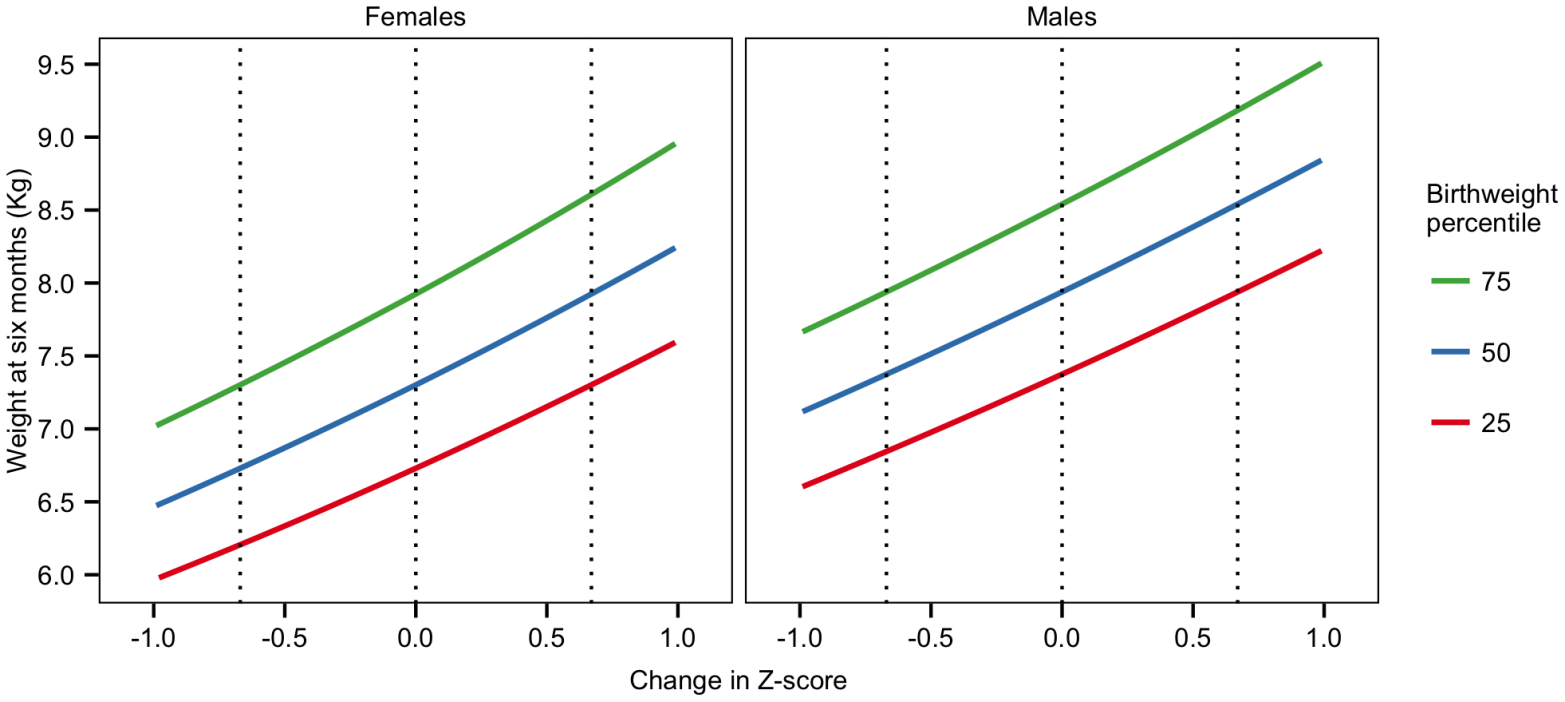
Theoretical description of the relationship between weight at six months and change in Z-score, as defined by the WHO growth curves. The relationship is displayed for multiple birthweight percentiles. A change in Z-score of 0 corresponds to theoretically perfect growth at six months. If a male child was born at the 75th percentile, then their expected weight at six months would be 8.5 kg (y-axis), corresponding to 0 change in Z-score (x-axis) on the right panel. If the child instead weighed 9.0 kg at six months (y-axis), then that would correspond to a 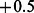 change in Z-score (x-axis).

The distribution of the difference in the estimated Z-scores was found to be approximately Normally distributed, with a mean of 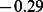, median of 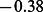, and IQR of 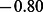 to 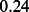 for females, and a mean of 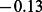, median of 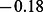, and IQR of 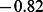 to 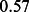 for males. To aid in the interpretation of Z-scores, the relationship (at different birth weights) between change in Z-score and weight at six months is displayed in Figure 1. Table 1 contains further descriptive characteristics of the study participants.

**Table 1:** Descriptive characteristics of study participants.

Characteristic	Description
Z–score change > 0.67 (%)	16.7
Z–score change < −0.67 (%)	32.2
Maternal smokers (%)	11.5
Twins (%)	3.3
Siblings (%)	61.8
Birthweight (Kg)	3.58 (3.27, 3.88)
Gestational age (days)	284 (277, 288)
Maternal age (yrs)	30 (28, 33)
Maternal BMI	24 (21, 26)
Sample size	218

Statistics are displayed as median (IQR) or only %. Sex specific results were not noticeably different from the above results.

### Exposures

The data in this study originated from a microarray dataset previously published [17]. The probes were constructed based on a limited 389 clone dataset [18] (constructed from DNA extracted from the fecal samples obtained on days 4, 10, 30, and 120) and subsequently evaluated on a 3845 clone dataset using Basic Local Alignment Search Tool (BLAST) [19] on a local database containing the dataset. Detailed information about this process can be found in a previous paper from the NOMIC study [17].

The exposures of interest are intensity readings for 22 probes, encoding different gut microbiota species (spp.) groups at 4, 10, 30, and 120 days since birth. The probes, labelling sequence, and target bacteria spp. groups are displayed in Table 2. The frequency of each probes detection, stratified by day and sex, are shown in the SI as Table S1.

**Table 2:** Probes and their targets.

#	Probe match	Labeling
1	*Enterococcus* spp.	TCATCCCTTGACGGTATCTAA
2	*Lactobacillus* spp.	GTCAAATAAAGGCCAGTTACTA
3	*Lactobacillus paracasei/casei*	CAGTTACTCTGCCGACCATT
4	*Staphylococcus* spp.	ACACATATGTTCTTCCCTAATAA
5	*Streptococcus* spp.	AGTGTGAGAGTGGAAAGTTCA
6	*Clostridium* spp.	TCAACTTGGGTGCTGCATTC
7	*Lachnospiraceae* spp.	AGCTAGAGTGTCGGAGAGG
8	*Veillonella* spp.	GATTGGCAGTTTCCATCCCAT
9	*Lachnospiraceae* spp.	TATCAGCAGGAAGATAGTGA
10	*Lachnospiraceae* spp.	AGTCAGGTACCGTCATTTTCT
11	*Lachnospiraceae* spp.	ACTGCTTTGGAAACTGCAGAT
12	*Pseudomonas* spp.	GTAGAGGGTGGTGGAATTTC
13	*Escherichia coli*	GAGCAAAGGTATTAACTTTACTC
14	Enterobacteriaceae other than *E. coli*	CGAAACTGGCAGGCTAGAGT
15	Gammaproteobacteria	CCTGGACAAAGACTGACGCT
16	*Varibaculum* spp.	TTGAGTGTAGGGGTTGATTAG
17	*Bifidobacterium longum* including subsp. *infantis*	GAGCAAGCGTGAGTAAGTTTA
18	*Bifidobacterium bifidum*	CCGAAGGCTTGCTCCCAAA
19	*Bifidobacterium breve*	CACTCAACACAAAGTGCCTTG
20	*Bifidobacterium* spp.	GCTTATTCGAAAGGTACACTCACCCCGAAGGG
21	*Bacteroides fragilis*	GGGCGCTAGCCTAACCAG
22	*Bacteroides* spp.	ATGCATACCCGTTTGCATGTA

The probe matches were taken from a previous paper [17].

Each intensity reading at every time point is dichotomised into either detected or non-detected. We selected this categorisation since we had no information on the distributions of the different probes' intensities in the average population, i.e. it was not possible to choose appropriate demarcations for low, moderate, or high levels.

Each microbiota spp. group were examined individually.

### Confounders and effect modification

Information on potential confounders was obtained by questionnaires filled in by the mothers and from the Medical Birth Registry of Norway. Variables considered *a priori* to be potential confounders were antibiotics use (after day 4 of life), sex, having received milk substitutes, maternal smoking, and parity, however, stepwise regression procedures led to the removal of all considered confounders due to a lack of effect.

After microbial exposures corresponding to altered growth were identified, the distributions of birthweight, usage of the newborn intensive care unit, preeclampsia, physician-diagnosed poor fetal growth as reported by the mother, gestational age, and maternal BMI were investigated with respect to microbial exposures, to identify if the findings could have been influenced by these variables. Usage of the newborn intensive care unit, preeclampsia, and poor fetal growth were not initially investigated as potential confounders due to their low prevalence in this subset, which prevented us from obtaining reliable effect estimates when including them in any model.

When considering the relationship between microbes and growth, our initial investigations found evidence for effect modification by sex. This led us to perform separate stratified analyses.

### Time-specific analyses

We were interested in identifying time points at which the detection of specific gut microbiota spp. groups were significantly associated with growth trajectory. That is, we investigated whether we could identify any time points, where the detection of gut microbiota spp. groups, shifted the growth outcome, the mean change in Z-score. We modelled this relationship by including the detection of gut microbiota at each time point (days 4, 10, 30, and 120) separately, using a standard linear regression model (separately for every gut microbiota spp. group). Thus the linear model constructed in our analysis is as follows:
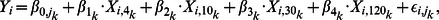
where 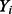 is the change in Z-score for the 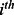 infant (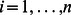), and 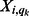 denotes the detection of the 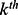 gut microbiota spp. group (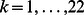) at the 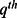 time point (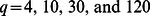).

We tested for the significance of 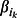 using the mixed directional false discovery rate (mdFDR) controlling method described in Guo et al. (2010) [20]. We first tested at significance level of 5%. We then repeated the analysis at 20% level of significance in order to identify biologically interesting results that were not statistically significant at the 5% level of significance. Briefly summarising the method, we defined 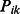 as the p-value for the test:
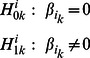
(1)for 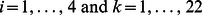. We then treated 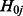 as the intersection of all 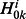 over 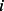, and 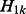 as the union of all 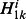 over 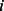. The following procedure was then undertaken:

1. The Benjamini-Hochberg method was applied at level 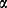 to test 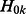 against 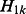 simultaneously for 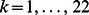, based on the Bonferroni pooled p-values 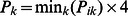

2. R denotes the total number of null hypotheses rejected.
3. All 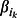 were tested, and 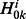 was rejected with adjusted significance level 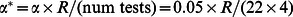

### Exposure patterns for a developing gut ecosystem

**Figure 2:**
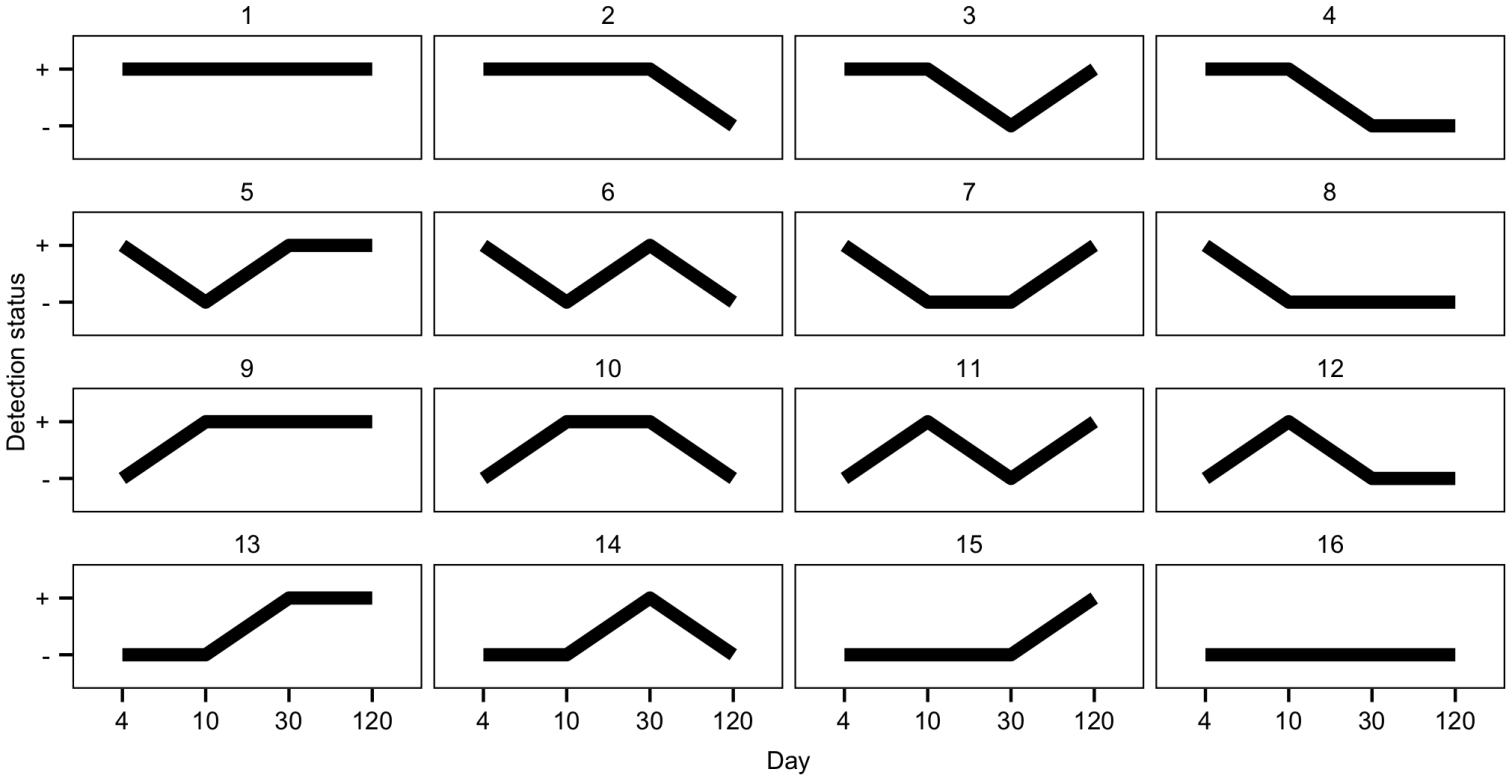
All possible exposure patterns in the data. **“** 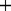 **” and “** 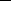 **” represent detection and non-detection respectively.** For example, pattern 8 indicates detection at day 4, followed by non-detection at days 10, 30, and 120.

It is conceivable that, in an infant, it is not the effect of the gut microbiota at a singular time point, but rather the gut ecosystem developing over time, which influences growth. To capture this evolution, it is possible to describe an infant's exposure to gut microbiota as a pattern over time. For example, one infant's pattern could be a gut microbiota spp. that is detected at days 4, 10, and 30, then non-detected at day 120. Each combination of possible values of the gut microbiota (detected or not detected) at different time points (4, 10, 30, 120 days) was considered to be a pattern. All 16 possible patterns are displayed in Figure 2.

If a pattern was observed to occur less than 15% of the time, it was not included as a testable pattern. Below 15% frequency we did not have adequate power to warrant testing. Let 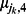 denote the population mean for the growth outcome variable (change in Z-scores, representing difference from expected growth) of infants with pattern 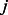, 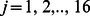 (where pattern 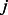 has 4 time points: days 4, 10, 30 and 120) for the 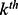 gut microbiota spp., 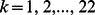. Let 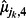 denote the estimate of 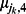 using the sample mean and let 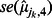 denote the standard error associated with the sample mean.

Using 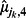 and 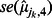 for each pattern and gut microbiota spp. group, we applied Tuke's method [21] to test for equivalence to zero:
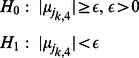
(2)where 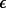 was chosen to be 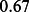, as mentioned previously. Our analysis focused on attempting to identify a pattern which corresponded to expected growth (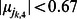) instead of a comparison analysis (e.g. pattern 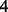 compared to pattern 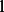 has an odds ratio of 2 for expected growth versus unexpected growth) for two reasons. Firstly, we believed that the former concept was more clinically useful and interesting than the latter. Secondly, we were unable to easily select an appropriate reference pattern (from those displayed in Figure 2) as multiple testing issues and concerns of model overfitting arose when considering multiple rounds of model fitting to identify the most appropriate reference patterns (e.g. the pattern with the most extreme growth).

In this analysis, we were concerned with identifying which gut microbiota spp. group patterns corresponded to a mean change in Z-score that was significantly close to zero (i.e. did not deviate from expected growth). This is in contrast to the previous time-specific analysis, which was focused on the relative shift in change in Z-score, when the exposure was either present of absent.

Similar to the previous analysis, we applied the mdFDR controlling method of Guo et al. (2010) at a rate of 0.05 [20]. Once we identified a significant pattern (i.e. one where 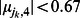), we tested to see if some time points might be superfluous and not adding information; for example, it may be that only the first 30 days of exposure that affect growth, so the last time point (day 120) would not be relevant and could be removed from the pattern. From the mixed directional false discovery rate controlling method [20], each four time point pattern was tested at an adjusted significance level of 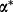; if the p-value of pattern 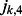 (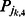) was less than 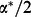 then (by using a Bonferroni adjustment) we had the opportunity to perform an additional test to pattern 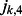 without risk of losing the significant result for the four time point pattern.

That is, consider the p-values of the patterns 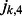, 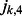 without day 120 (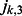), 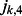 without days 30 and 120 (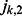), and 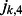 without days 10, 30, and 120 (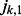), to be denoted as 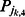, 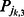, 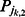, and 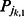, respectively. The following procedures were performed after finding a four time point pattern 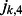 whose mean is significantly close to zero:

1. If 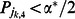, then 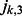 was tested at significance level 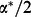

2. If 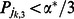, then 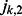 was tested at significance level 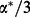

3. If 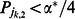, then 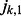 was tested at significance level 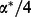

The process ended when a pattern's mean was either not significantly close to zero, or when 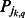 (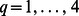) was not large enough to allow continued testing. This process controlled the false discovery rate, while simultaneously ensuring that no significant finding was subsequently lost by the additional testing to remove superfluous time points. A short proof, that this adaptation still retains control of the false discovery rate, is provided in the SI. By implementing this adaptation, the resultant hypotheses of interest were:
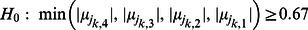


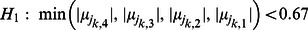
The data reduction process was only considered from the right side of the pattern to avoid confounding. By definition, a confounder must affect both the exposure and outcome, and it is not possible for an exposure at day 120 to affect the exposure between days 4 and 30. In contrast, an exposure at day 4 may influence the exposure at day 10, and is therefore a possible confounder. We stress that, by only undertaking this process on the right side of the pattern, we do not imply that the right side of the pattern is less important. Instead, we view the process as adding information where possible (by culling superfluous points on the right side of the pattern) and leaving the pattern otherwise alone.

### Post-hoc screening of results

If a pattern was found to have its mean significantly close to zero (i.e. the null hypothesis in (2) is rejected), the mean of the pattern's crude contrast (i.e. if detection at days 4 and 10 was significant, the crude contrast would be non-detection at days 4 and 10) was tested for difference to zero, using a t-test at 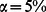. If the crude contrast was not found to be significantly different from zero, the pattern was discarded from the significant findings. In the event of a significant crude contrast, a Welch two sample t-test was performed to test if the means of the pattern and crude contrast differed from each other. This test was performed at a significance level of 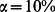 due to the decrease in sample size (and hence power) when only considering the set of infants with either the pattern of interest or the crude contrast. Tests found to be significant at 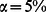 were noted as such.

## Results/Discussion

**Figure 3:**
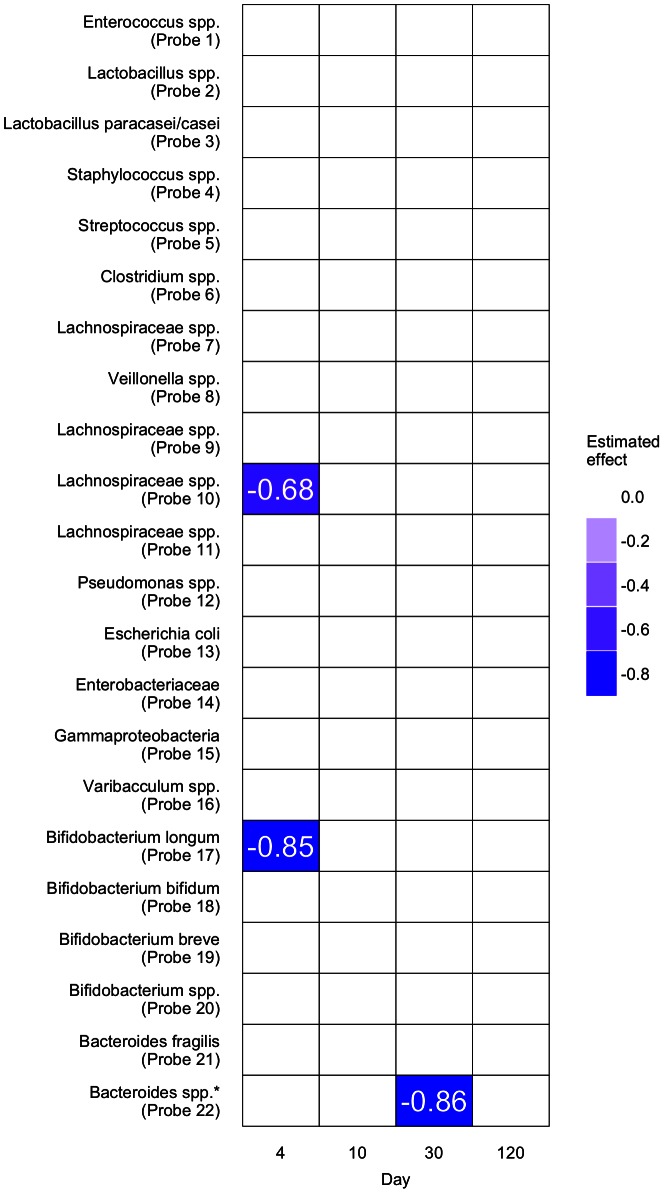
Results from the time-specific analyses for males. Coloured areas indicate significant results at 20% false discovery rate, and are labelled with their effect estimates, while white areas indicate non-significant results. Significant results at 5% false discovery rate are indicated by 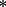. Only the results for males are displayed, as no significant results were found for females.

**Figure 4:**
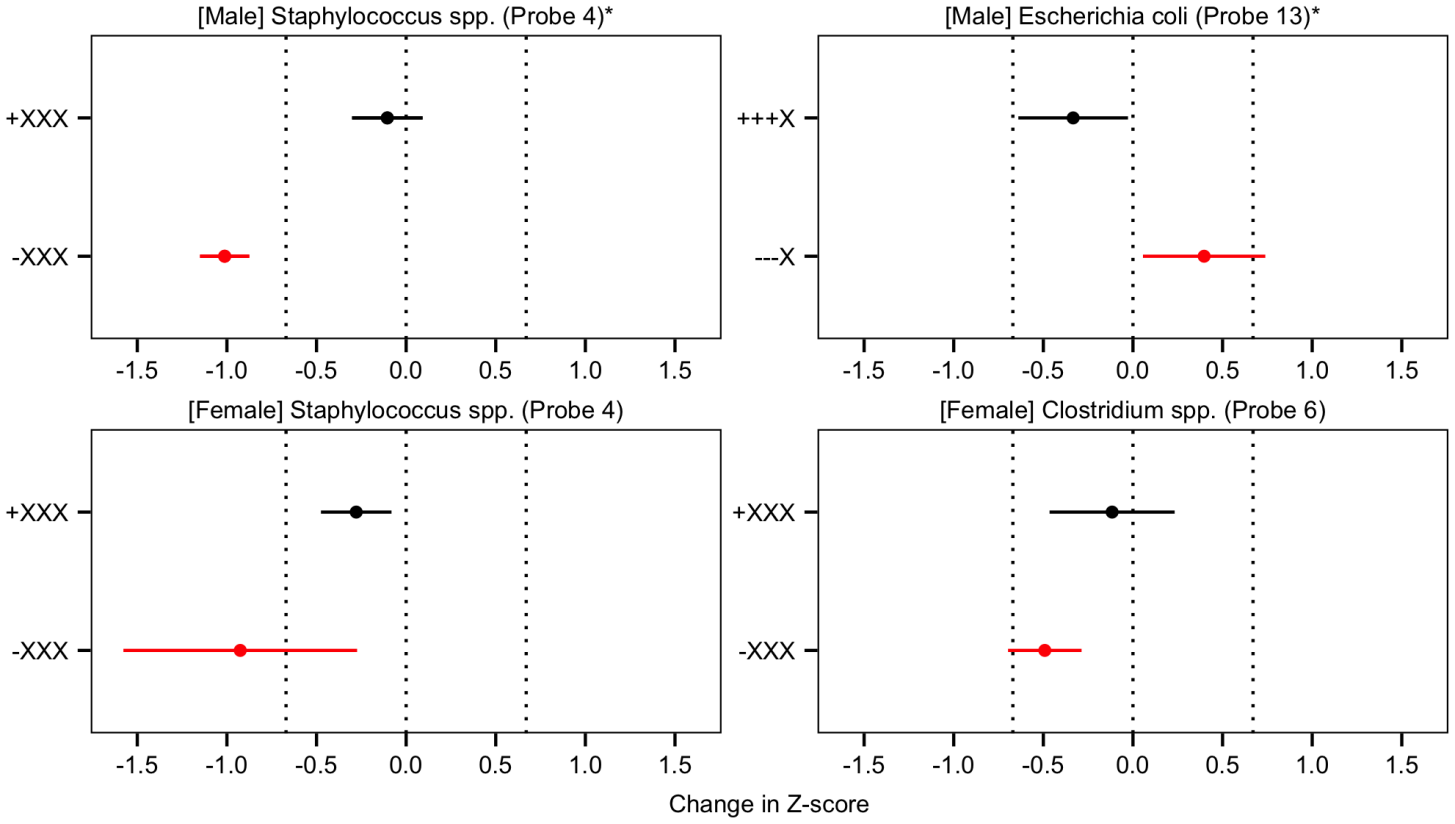
Results from the pattern analysis. The exposure pattern is represented by four characters, constructed from “
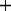
”, “
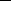
”, and “X”, which represent detection, non-detection, and irrelevance, respectively, for the four time points of the analysis (days 4, 10, 30, and 120). The black points and lines represent estimated means and 95% confidence intervals for patterns that were found to be significantly close to zero at an false discovery rate of 5%. The crude contrasts (i.e. if “
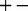
XX” was significant, the crude contrast would be “
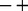
XX”) that were significantly different to zero at 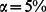 have their estimated means and 95% confidence intervals displayed in red. For the testing of the difference of the means of the two patterns, significant results (at 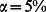) is indicated by 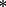, otherwise significance is 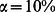. Vertical lines are displayed at 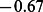 and 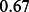, representing the boundaries of expected growth.

We applied the methods (listed above) to each gut microbiota spp. group in Table 2 and displayed significant time-specific results (from standard linear regressions) in Figure 3 and pattern results (from our novel method) in Figure 4.

In the time-specific analyses, with a false discovery rate of 5% applied, we found the detection of *Bacteroides spp.* (Probe 22) at day 30 to be significantly associated with reducing growth in males, when compared to non-detection (Figure 3). The current literature shows that *Bacteroides spp.* is protective against obesity [22].

In the pattern analyses, we note that the detection of *Staphylococcus* spp. (Probe 4) at day 4 was associated with expected growth in females and males (Figure 4). 98 males (96%) and 94 females (91%) had detectable levels of *Staphylococcus* spp. (Probe 4) at day 4. The literature highlights that colonisation of *Staphylococcus* spp. is a normal feature of healthy gut flora [23]. We also found that *Escherichia coli* (Probe 13) detection from day 4 through to 30 was associated with expected growth in males (Figure 4), which occurred in 75 (77%) of the males. The current literature indicates that colonisation of *Escherichia coli* is a normal feature of healthy gut flora development [24].

We are careful to refer to our exposures as “detected” and “not-detected”, and never as “present” and “absent”. This is because our detection limits for the different bacteria are likely very different, and therefore such references would be inappropriate, even though we are using detected/not-detected as a proxy for present/absent. Higher (and varying) detection limits results in misclassification of the exposure, and biases our results towards the null. This does not invalidate our findings, but did reduce our ability to identify additional significant findings.

We were concerned that our pattern analysis findings were caused by confounding that occurred before four days of life. When comparing infants with detected *Staphylococcus* spp. (Probe 4) at day 4 to those without, we found evidence that males with non-detected *Staphylococcus* spp. (Probe 4) at day 4 had lower birthweight (mean 3.19 Kg vs 3.58 Kg) and higher proportion of usage of the newborn intensive care unit, (25% vs 6%), however, these findings were inverted in the female stratum (3.68 Kg vs 3.55 Kg and 0% vs 5%), and we therefore found no conclusive evidence of confounding. We also found no noticeable differences in the rates of preeclampsia, poor fetal growth, gestational age, or maternal BMI. No noticeable differences were found in any of the above variables when checking for confounding in *Escherichia coli* (Probe 13).

While our outcome was focused on investigating growth in the first six months of life, the growth rate of a child is set in different phases during life, including in utero [16]. As we found no evidence of confounding by physician-diagnosed poor fetal growth, we were not concerned with issues pertaining to in utero growth. While we had data on growth at 6, 12, and 24 months, we ultimately chose approximately 6 months as the outcome as we had the most complete dataset at this time, and reviews on rapid growth in early childhood failed to differentiate any time point between 6 to 24 months as clearly superior [13], [16].

Confounding by race and ethnicity was not considered due to the low proportion of non-ethnically Norwegian mothers in the study (11% in the subset used for the analysis). Duration of exclusive breastfeeding was evaluated with our surrogate variable, “use of milk substitutes.” This was not found to be an important confounder, probably due to the long length of average breastfeeding in Norway (greater than one year; only 33% of the subset used for the analysis had used one or more milk substitutes before day 30).

By investigating one overarching theme (“how does the gut microbiota affect infant growth?”) through two different questions, we obtained two different set of results. We note that these two set of results are not mutually exclusive, nor contrasting in nature. Instead they offer different perspectives: the time-specific analysis aids in highlighting where gut microbiota has an association with the mean of the outcome, which is useful in situations where the outcome is shifted away from 0 and it is hard to find a true “healthy reference group”. The pattern analysis is useful in identifying how the gut microbiota develops over time in babies with expected growth (i.e. we found that *Escherichia coli* (Probe 13) detection from day 4 through to 30 was associated with expected growth in males). This allowed us to combine a number of exposures over time, which, when viewed together, formed a cohesive message about the outcome. The message was that certain patterns corresponded to expected growth, and deviation from those patterns was associated with not achieving expected growth – instead of only identifying singular gut microbiota exposures that shifted growth.

It is important to note that as no contrasts (beyond the crude contrasts) were compared to the “expected growth” pattern, we cannot make inferences about the association between expected growth and patterns that are partially different from the “expected growth” pattern. We can only assert that the presence of particular exposure patterns are associated with expected growth, and that they significantly differed from their crude contrasts (which were also significantly different from expected growth).

When considering the application of the pattern analysis method to other analyses, it is important to note that it cannot account for confounding. We propose that in situations where confounding variables are at work, the above method be used to extract a plausible reference pattern, and then a traditional logistic regression strategy should be implemented to address confounding. This process of reference pattern selection adds value to the current methodology literature, as it enables the transparent selection of a sensible reference pattern in scenarios (such as the one above) where it is not a simple matter to select a baseline *a priori*. In addition, other analyses may contain a multitude of time points, which would make the current strategy of creating longitudinal patterns unfeasible. In such situations, it would be advisable to “bin” similar time points to reduce the complexity of the dataset, and then apply our procedure in an attempt to identify binned time points that are interesting. Once such binned time points are identified, the method can be reapplied in the original data, restricted to the time points of interest. There are no issues with including more taxa, as the method is applied to each taxon independently. In addition, reference patterns are only considered when they have high frequencies and abnormal conditions are by definition less common. It is therefore necessary to “search” for a healthy common reference pattern and then test to see if deviating from the healthy reference (i.e. the crude contrast) results in illness.

In certain situations, the outcome may be dependent on the interaction between two gut microbiota spp. groups, which would result in the above method not being appropriate without an extension. By creating patterns consisting of two – or more – gut microbiota spp. groups, and then applying the methods described here, the intra-gut microbiota spp. group dependencies can be accounted for.

As with all methods, we are limited by the granularity of our longitudinal observations and the observational nature of our data. Our method identifies time-dependent points that may contain information about potential time-dependent exposure windows that are reflected in the observed data. That is, if one assumes there is a time-dependent exposure window requiring a microbe to be detected between 100–110 days, but the microbe does not simply dissipate from the body at day 111, so a strong relationship exists between day 110 and 120, then the method will identify a time-dependent point at day 120 (reflecting the time-dependent exposure window at days 100–110). This is simply a feature of the data, and the length of time surrounding each time-dependent exposure window when it is reflected in the data (i.e. when the microbes remain similar) may vary from microbe to microbe and be dependent on the situation at hand.

The only way to prove that a time-dependent exposure window has occurred is through experiments. Using observational data, our method provides a novel way to describe potential time-dependent exposure windows that may have been reflected into the observable data. These descriptions can be further used to create time-dependent hypotheses for experiments concerned with the existence of time-dependent exposure windows. Furthermore, we highlight that our statistical methods were designed to control the false discovery rate, over a large number of tests. In doing so, it is likely that we discarded a number of clinically significant findings that were not found to be statistically significant. We therefore make no claims about the gut microbiota spp. groups that were not found to have any significant results, as the absence of evidence is not evidence of absence.

Our outcome (the difference in Z-scores of weight-for-age for approximately 6 months versus birth) was not centred around 0 (mean/median of 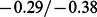 and 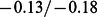 for females and males respectively), which raised concerns that our weight-for-age variable was perhaps inappropriate, and that a measure that also included length might be more appropriate. We investigated the larger Norwegian Human Milk Study cohort (n = 3529), of which NOMIC is a subsample [25]. We found that the median weight-for-age Z-score at birth was 0.76, decreasing to 0.31 at approximately 6 months of life, while the median weight-for-length Z-score at birth was 0.63, decreasing to 0.06 at approximately 6 months. This suggests that the Norwegian infants were born with more mass than one would expect for their appropriate length, and both weight-for-age and weight-for-length measures show similar trends.

These findings from the larger Norwegian Human Milk Study cohort were similar to what we found in NOMIC. Similar results have been shown in the Norwegian Medical Birth Registry, where it has been found that from the early 1970s to the late 1990s the birthweight of Norwegian infants has been increasing [26]. These findings strengthen the recommendations from the Norwegian Health Directorate to use the World Health Organization's growth curves [14]. It is also worth noting that because the female distribution is centred so far from zero (mean/median of 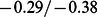), we lack power when detecting gut microbiota patterns that results in a positive change in Z-score. Furthermore, our “approximately 6 months” Z-score was calculated as the closest observed Z-score to 6 months, within 4–8 months. This implies an inherent assumption that the growth velocity of the child at the observed time point (between 4–8 months) is not different to that of the child at 6 months. This assumption may over or under-estimate the growth velocity, however, it will do so entirely at random (with regards to the exposure) and therefore will only bias the results towards the null, and does not invalidate our findings.

Our results expand on the current literature relating gut microbiota to growth, in both methodology and biological findings. With regards to methodology, we developed a novel method to analyse longitudinal data that contains information about the development of an ecosystem over time. Crucially, this method controls the false discovery rate associated with multiple levels of multidimensional testing. We expanded the biological literature by reporting time-dependent patterns associated with expected growth, which, in some cases, confirmed the importance of gut microbiota spp. groups previously reported on.

## Supporting Information

Table S1 **Probes and the frequency of their detection.** The frequency of the detection of each probe, stratified by sex and day. Information is presented as percent detected.
(PDF)

Text S1 **Analytical discussion of the mdFDR control.**
(PDF)
