# Supplementary material for: Novel Developmental Analyses Identify Longitudinal Patterns of Early Gut Microbiota that Affect Infant Growth
Source: PLoS Comput Biol. 2013 May 9;9(5):e1003042. doi: 10.1371/journal.pcbi.1003042 (PMC3650000; doi:10.1371/journal.pcbi.1003042)
Supplement: Table S1 — Probes and the frequency of their detection. The frequency of the detection of each probe, stratified by sex and day. Information is presented as percent detected. (PDF) [file pcbi.1003042.s001.pdf]

Table S1. Probes and the frequency of their detection

#	Probe match	Female Day 4	Female Day 10	Female Day 30	Female Day 120	Male Day 4	Male Day 10	Male Day 30	Male Day 120
1	*Enterococcus* spp.	56	51	54	66	64	51	54	62
2	*Lactobacillus* spp.	40	47	50	36	25	37	44	34
3	*Lactobacillus paracasei/casei*	49	59	60	61	62	55	61	72
4	*Staphylococcus* spp.	91	98	84	60	96	94	83	58
5	*Streptococcus* spp. (α-hemolytic)	46	54	48	25	49	45	54	36
6	*Clostridium* spp.	42	42	43	55	40	40	48	69
7	*Lachnospiraceae* spp.	27	26	34	51	29	28	26	54
8	*Veillonella* spp.	30	52	68	67	28	48	71	66
9	*Lachnospiraceae* spp.	48	49	53	67	44	48	56	70
10	*Lachnospiraceae* spp.	48	49	59	75	53	60	62	72
11	*Lachnospiraceae* spp.	63	57	65	61	60	64	59	68
12	*Pseudomonas* spp.	68	71	70	57	71	72	66	64
13	*Escherichia coli*	62	63	60	80	63	67	66	72
14	Enterobacteriaceae other than *E. coli*	75	81	81	88	86	89	82	89
15	Gammaproteobacteria	86	91	94	98	92	95	91	94
16	*Varibaculum* spp.	37	29	36	39	31	31	27	51
17	*Bifidobacterium longum* including subsp. *infantis*	84	81	80	84	84	84	83	87
18	*Bifidobacterium bifidum*	82	79	83	88	73	81	83	86
19	*Bifidobacterium breve*	50	55	59	63	39	48	54	66
20	*Bifidobacterium* spp.	79	80	84	83	74	76	77	79
21	*Bacteroides fragilis*	35	30	35	31	29	27	30	19
22	*Bacteroides* spp.	68	47	63	61	52	47	48	45
	(Sample size)	103	105	103	100	102	100	103	100

The frequency of the detection of each probe, stratified by sex and day. Information is presented as percent detected.
