# Supplementary material for: Novel Developmental Analyses Identify Longitudinal Patterns of Early Gut Microbiota that Affect Infant Growth
Source: PLoS Comput Biol. 2013 May 9;9(5):e1003042. doi: 10.1371/journal.pcbi.1003042 (PMC3650000; doi:10.1371/journal.pcbi.1003042)
Supplement: Text S1 — Analytical discussion of the mdFDR control. (PDF) [file pcbi.1003042.s002.pdf]

Text S1

For $k = 1, \ldots, 22$ gut microbiota spp. groups with $j = 1, \ldots, 16$ patterns, let the p-values of the patterns $j_{k,4}$, $j_{k,4}$ without day 120 ($j_{k,3}$), $j_{k,4}$ without days 30 and 120 ($j_{k,2}$), and $j_{k,4}$ without days 10, 30, and 120 ($j_{k,1}$), be denoted as $P_{j_{k,4}}$, $P_{j_{k,3}}$, $P_{j_{k,2}}$, and $P_{j_{k,1}}$, respectively (obtained using Tuke's method). These patterns are considered to be part of the pattern family j_k. We aim to test the following:

$$H_0 : \min\left(|\mu_{j_{k,4}}|, |\mu_{j_{k,3}}|, |\mu_{j_{k,2}}|, |\mu_{j_{k,1}}|\right) \geq 0.67$$
$$H_1 : \min\left(|\mu_{j_{k,4}}|, |\mu_{j_{k,3}}|, |\mu_{j_{k,2}}|, |\mu_{j_{k,1}}|\right) < 0.67$$

which corresponds to

$$H_0 : \left\{|\mu_{j_{k,4}}| \geq 0.67\right\} \cap \left\{|\mu_{j_{k,3}}| \geq 0.67\right\} \cap \left\{|\mu_{j_{k,2}}| \geq 0.67\right\} \cap \left\{|\mu_{j_{k,1}}| \geq 0.67\right\}$$
$$H_1 : \left\{|\mu_{j_{k,4}}| < 0.67\right\} \cup \left\{|\mu_{j_{k,3}}| < 0.67\right\} \cup \left\{|\mu_{j_{k,2}}| < 0.67\right\} \cup \left\{|\mu_{j_{k,1}}| < 0.67\right\}$$

Given an arbitrary significance level α^*, the following procedures are performed after finding a four time point pattern $j_{k,4}$ whose mean is significantly close to zero:

1. If $P_{j_{k,4}} < \alpha^*/2$, then $j_{k,3}$ is tested at significance level $\alpha^*/2$
2. If $P_{j_{k,3}} < \alpha^*/3$, then $j_{k,2}$ is tested at significance level $\alpha^*/3$
3. If $P_{j_{k,2}} < \alpha^*/4$, then $j_{k,1}$ is tested at significance level $\alpha^*/4$

The Bonferroni adjusted p-value for gut microbiota spp. group k is denoted as

$$P_k = (\text{num patterns tested in gut microbiota spp. group } k) \times min_{j \in k}(P_{j_{k,4}})$$

Without loss of generality, we will prove that the FDR is controlled when step 1 is implemented - proofs for the other steps are similarly formed. This proof relies on the proof provided in Guo et al. (2010). We let V be the number of false positives, $I_1 = \{1 \leq k \leq 22 | H_1\}$ be the set of indices of false null hypotheses, $\alpha^* = \alpha \times R/(22 \times 16)$ (obtained from Guo et al., 2010), and $I(\cdot)$ be an indicator function. We then

express V as:

$$
\begin{aligned}
V &= \sum_{k\in I_1} I\left(\cup_{j=1}^{16} (\text{Reject } H_0 \text{ for } j_k|H_0)\right) \\
&= \sum_{k\in I_1} I\left(\cup_{j=1}^{16} \left(\left(\alpha^*/2 \le P_{j_{k,4}} \le \alpha^*|H_0\right) + \left(P_{j_{k,4}} \le \alpha^*/2, P_{j_{k,3}} \le \alpha^*/2|H_0\right)\right)\right)
\end{aligned}
$$

Then

$$
\begin{aligned}
FDR =& E\left\{\frac{V}{R \vee 1}\right\} \\
=& E\left\{\frac{E(V|R=r)}{R \vee 1}\right\} \\
=& E\left[\frac{\sum_{j\in I_1} \Pr\left\{\cup_{j=1}^{16} \left(\left(\alpha^*/2 \le P_{j_{k,4}} \le \alpha^*|H_0\right) + \left(P_{j_{k,4}} \le \alpha^*/2, P_{j_{k,3}} \le \alpha^*/2|H_0\right)\right) |R=r\right\}}{R \vee 1}\right] \\
=& \sum_{r=1}^{22} \sum_{k\in I_1} \frac{1}{r} \Pr\left\{\cup_{j=1}^{16} \left(\left(\alpha^*/2 \le P_{j_{k,4}} \le \alpha^*, R=r|H_0\right) + \left(P_{j_{k,4}} \le \alpha^*/2, P_{j_{k,3}} \le \alpha^*/2, R=r|H_0\right)\right)\right\} \\
\le& \sum_{r=1}^{22} \sum_{k\in I_1} \sum_{j=1}^{16} \frac{1}{r} \left\{\Pr\left(\alpha^*/2 \le P_{j_{k,4}} \le \alpha^*, R=r|H_0\right) + \Pr\left(P_{j_{k,4}} \le \alpha^*/2, P_{j_{k,3}} \le \alpha^*/2, R=r|H_0\right)\right\}
\end{aligned}
$$

The above inequality follows from the Bonferroni inequality

$$
\begin{aligned}
\le& \sum_{r=1}^{22} \sum_{k\in I_1} \sum_{j=1}^{16} \frac{1}{r} \left\{\Pr\left(\alpha^*/2 \le P_{j_{k,4}} \le \alpha^*, R=r|H_0\right) + \right. \\
& \left. max\left(\Pr\left(P_{j_{k,4}} \le \alpha^*/2, R=r|H_0\right), \Pr\left(P_{j_{k,3}} \le \alpha^*/2, R=r|H_0\right)\right)\right\} \\
=& \sum_{r=1}^{22} \sum_{k\in I_1} \sum_{j=1}^{16} \frac{1}{r} \left\{\Pr\left(\alpha^*/2 \le P_{j_{k,4}} \le \alpha^*|H_0\right) \times \Pr\left(R^{(-k)} = r-1\right) + \right. \\
& \left. max\left(\Pr\left(P_{j_{k,4}} \le \alpha^*/2|H_0\right), \Pr\left(P_{j_{k,3}} \le \alpha^*/2|H_0\right)\right) \times \Pr\left(R^{(-k)} = r-1\right)\right\}
\end{aligned}
$$

The above simplification results from the assumption that each gut microbiota spp. group is independent of each other, where $R^{(-k)}$ denotes the number of rejections in the step up procedure with critical constants $a_l = \dfrac{l+1}{22}, l = 1, \ldots, 22-1$ based on $\{P_1, \ldots, P_{22}\} \backslash \{P_k\}$

$$
\le \sum_{r=1}^{22} \sum_{k\in I_1} \sum_{j=1}^{16} \frac{1}{r} \left\{\alpha^*/2 \times \Pr\left(R^{(-k)} = r-1\right) + max\left(\alpha^*/2, \alpha^*/2\right) \times \Pr\left(R^{(-k)} = r-1\right)\right\}
$$

The above inequality follows from $\Pr(pvalue \le p) \le p,$ for any $p \in (0,1)$ under H_0

$$
\begin{aligned}
&= \sum_{r=1}^{22} \sum_{k \in I_1} \sum_{j=1}^{16} \frac{1}{r} \left\{ \alpha^* \times \Pr\left(R^{(-k)} = r-1 \right) \right\} \\
&= \sum_{r=1}^{22} \sum_{k \in I_1} \sum_{j=1}^{16} \frac{1}{r} \left\{ \alpha \times R/(22 \times 16) \times \Pr\left(R^{(-k)} = r-1 \right) \right\} \\
&= \frac{m_1}{22} \alpha \\
&\leq \alpha
\end{aligned}
$$
